# Progress and prospects of biomarker-based targeted therapy and immune checkpoint inhibitors in advanced gastric cancer

**DOI:** 10.3389/fonc.2024.1382183

**Published:** 2024-06-14

**Authors:** Zhu Zeng, Qing Zhu

**Affiliations:** Department of Abdominal Oncology, West China Hospital, Sichuan University, Chengdu, China

**Keywords:** gastric cancer, signaling pathway, HER-2, c-MET, Claudin 18.2, immune checkpoint inhibitors, biomarkers

## Abstract

Gastric cancer and gastroesophageal junction cancer represent the leading cause of tumor-related death worldwide. Although advances in immunotherapy and molecular targeted therapy have expanded treatment options, they have not significantly altered the prognosis for patients with unresectable or metastatic gastric cancer. A minority of patients, particularly those with PD-L1–positive, HER-2–positive, or MSI-high tumors, may benefit more from immune checkpoint inhibitors and/or HER-2–directed therapies in advanced stages. However, for those lacking specific targets and unique molecular features, conventional chemotherapy remains the only recommended effective and durable regimen. In this review, we summarize the roles of various signaling pathways and further investigate the available targets. Then, the current results of phase II/III clinical trials in advanced gastric cancer, along with the superiorities and limitations of the existing biomarkers, are specifically discussed. Finally, we will offer our insights in precision treatment pattern when encountering the substantial challenges.

## 1 Introduction

Gastric cancer (GC) is increasingly recognized as a major global healthcare issue, swiftly becoming a leading cause of cancer-related deaths worldwide (1, 2). It was estimated that, each year, over one million are newly diagnosed GC cases (3). Chronic infection with *Helicobacter pylori* (*H. pylori*), tobacco intake, alcohol consumption, and a high-salt diet together constitute genetic risk factors for GC (4–6). Often, radical resection is not available at diagnosis, primarily attributing to a significant number of patients present with unresectable or metastatic GC/gastroesophageal junction cancer (GEJC) (7). As a result, the majority could receive the systematic treatments based on conventional chemotherapy (7, 8). Yet, paradoxically, the overall 5-year survival rate remains below 10%. Novel approaches including targeted therapy and immunotherapy have emerged due to limited efficacy of traditional chemotherapy regimens. In the realm of precision medicine, the approach to treating advanced GC has undergone a substantial evolution, progressively steering toward personalized treatment pattern (9). This shift reflects the growing emphasis on precision and individualization in oncology.

During GC progression, multiple signaling pathways and molecular biological processes are involved. Common mutations occur in *TP53* and *CDH1* genes in GC. Additionally, DNA methylation of the *MLH1* gene correlates closely with microsatellite instability. Furthermore, signaling pathways such as the epidermal growth factor receptor (EGFR), the mitogen-activated protein kinase (MAPK), and the human epidermal growth factor receptor 2 (HER-2) signaling pathways, along with their crosstalk, contribute to cell growth, differentiation, and migration in GC (10, 11). Another crucial axis, the vascular endothelial growth factor (VEGF)/VEGF receptor (VEGFR), is recognized as a pivotal mediator in tumor angiogenesis (12). Blocking VEGF/VEGFR signal directly affects vascularization and even reverse the immune-suppressive tumor microenvironment (TME) by reducing the infiltration of regulatory T cells (Tregs) and so on (13).

In the treatment of advanced GC, targeting HER-2 signaling pathway is feasible in patients with advanced GC with HER-2–positive; meanwhile, the addition of immunotherapy is also recommended (14, 15). However, only about 10.4%–20.2% of patients with GC are HER-2–positive (16). This highlights an urgent need for novel, targeted therapies, particularly for those with HER-2–negative GC. As mentioned previously, the current evidence has distinguished patients who are especially responsive to immune checkpoint inhibitors (ICIs), including those with high-expression programmed cell death ligand 1 (PD-L1), or with Microsatellite instability high (MSI-H)/deficient mismatch repair protein (dMMR), or with Epstein–Barr virus infection (17–21). They are identified as the most suitable candidates and best-responders to ICIs. Moreover, emerging therapeutic targets such as Claudin 18.2 and cellular–mesenchymal-epithelial transition factor (c-MET) are gaining attention in the field (22–24). Despite the existing advances, the intricate roles and interactions among distinct signaling pathways, as well as the complex networks of multi-biomarkers informed by molecular features and genomic heterogeneity, remain largely elusive. To facilitate the optimization of treatment strategies in GC, we synthesize the latest findings from in-depth trials and further shed light on the future perspectives in this review.

## 2 Molecular targeted therapy in gastric cancer

**Figure 1:**
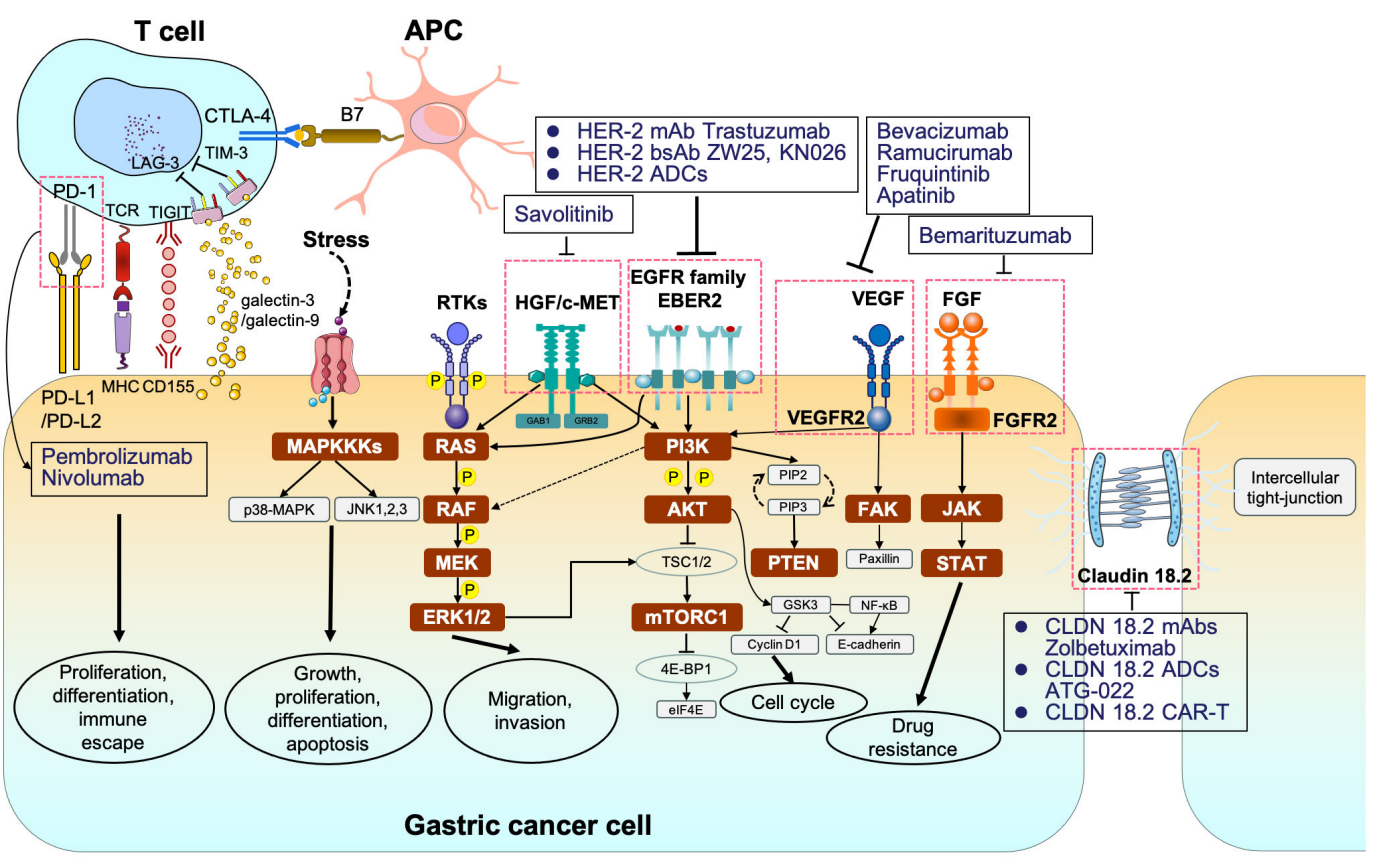
The signaling pathways and corresponding molecular targeted therapy in gastric cancer. MAPKKK, mitogen-activated protein kinase kinase kinases; p38-MAPK, p38 group of mitogen-activated protein kinases; JNK, jun amino-terminal kinase; RTKs, receptor tyrosine kinases; RAS, rat sarcoma; RAF, rapidly accelerated fibrosarcoma; MEK, mitogen-activated protein kinase; ERK1/2, extracellular signal–related kinase 1/2; PI3K phosphoinositide 3-kinase; AKT, protein kinase B; TSC1/2, tuberous sclerosis complex 1/2; mTORC1/2, mammalian target of rapamycin complex 1/2; 4E-BP1, eukaryotic translation initiation factor 4E (eIF4E)–binding protein 1; STAT, signal transducers and activators of transcription.

Evidence suggested that the occurrence and invasion of GC is driven by complicated signaling webs, not only attributing to a single factor (**Figure 1**; **Table 1**). Unfortunately, despite the complexity and diversity of signal networks, insights on these molecules have not yet been translated as targetable into the clinical practice. The most maturely studied target refers to HER-2. To better understand the mechanism of related signaling axis and to identify more novel but promising target, we will introduce the regulatory role of different pathways and further discuss the current implications of pathway-based targeted agents in unresectable or metastatic GC.

**Table 1:** The role of multiple signaling pathways in gastric cancer.

Signaling pathway	Function	Potential target
MAPK	Cell growth, cell differentiation, migration, tumor invasion, cell apoptosis	RTKs, ERK, JNK, p38-MAPKs, MEK, RAS, RAF
HER-2	Cell proliferation, cell differentiation, migration, angiogenesis	EGFR, HER-2/3/4, ERK, PTEN
PI3K/AKT/mTOR	Cell proliferation, migration, cell cycle, apoptosis, angiogenesis	RTKs, PI3K, AKT, mTOR, PTEN, TSC1/2, mTORC1/2, GSK3, PDK1
VEGF/VEGFR	Angiogenesis, immune response	VEGF, VEGFR-2
Wnt/β-catenin	Cell proliferation, cell differentiation, cell cycle, apoptosis, migration, immune response	TCF4, Gpx4
FGFR	Cell proliferation, cell differentiation, migration, angiogenesis	JAK, YAP
EGFR	Protein synthesis, cell growth, cell differentiation, migration	PI3K, AKT, EIF4E
HGF/c-MET	Cell proliferation, migration, cell cycle, apoptosis, hypoxia, inflammation	CXCL12, CXCR4
PD-1/PD-L1	Cell proliferation, apoptosis, immune response	PD-L1, PD-L2, TIM-3, LAG-3, TIGHT
CTLA-4/B7	Immune response	CTLA-4, B7

### 2.1 The role of MAPK signaling pathway

The MAPK family is a large serine kinase that could comprise five components, known as the extracellular signal–related kinases 1/2 (ERK1/2), ERK3/4, ERK5, c-Jun N-terminal kinase (JNK), and p38-MAPK, respectively (11, 25–27). The activation of the MAPK signaling pathway is typically initiated by the stimulation of upstream RAS proteins, which undergo a conformational shift in guanine triphosphatases, including Kirsten rat sarcoma viral oncogene homolog (KRAS), Harvey rat sarcoma viral oncogene homolog (HRAS), and Neuroblastoma rat sarcoma viral oncogene homolog (NRAS) (28–30). Then, the RAF proteins (such as Proto-oncogene serine/threonine-protein kinase (ARAF), vrafmurine sarcoma viral oncegene homolog B (BRAF), and Raf-1 proto-oncogene serine/threonine-protein kinase (CRAF)) are activated after phosphorylation, which, in turn, facilitates sequential interactions with downstream effector proteins, culminating in the formation of the classical RAS/RAF/MEK/ERK pathway (31, 32).

Given the in-depth research in GC, the MAPK/ERK signaling is involved in regulating various cellular biological functions via three core kinases (MAPKKKs, MAPKKs, and MAPKs) (33, 34). For instance, the matrix metalloproteinases (MMPs) have been identified as crucial factors associated with the invasion and migration of GC cells (35). Upstream elements of the MAPK/ERK pathway, such as interleukin-22 (IL-22), RAS protein activator like 1 (RASAL1), and nuclear apoptosis-inducing factor 1 (NAIF1), are involved in mediating cell migration and adhesion by regulating MMP activity (35, 36). Spondin-2 (SPON2), a member of the Mindin-F-spondin family, has been linked to metastasis in GC, particularly when it is highly expressed (37, 38). Numerous studies have demonstrated that SPON2 can promote the epithelial-mesenchymal transition (EMT) of GC cells by activating the MAPK/ERK signaling pathway, thereby accelerating metastasis (38, 39). In addition, the JNK module targets the activator protein-1 (AP-1) transcription factor, playing a vital role in GC cell proliferation and apoptosis (40). Furthermore, the p38-MAPK/AP-1 pathway has been identified as a significant factor associated with chemotherapy resistance in human GC cells (40, 41).

### 2.2 The role of HER-2 signaling pathway

HER-2, a proto-oncogene, belongs to the EGFR family of proteins, which is composed of HER-1 (ErbB1 and EGFR), HER-2 (ErbB2 and NEU), HER-3 (ErbB3), and HER-4 (ErbB4) (10, 42). HER-2 can form either homologous or heterologous dimers with HER-1 or HER-3 through dimerization to directly triggering its downstream signal cascades (RAS/RAF/MEK/ERK and PI3K/AKT pathway included), thereby promoting cell proliferation and migration (42–44). However, the specific ligands of HER-2 protein remain unclear. Evidence suggested that HER-2, when coupled with HER-3, exhibits a heightened activation potential (42). Abnormalities in HER-2, often owing to gene amplification and mutation, are implicated in various oncogenic processes (45). Amplification typically leads to an increase in copy numbers, resulting in the overexpression of the HER-2 protein, which is the most common type observed in solid tumors (such as breast cancer, GC, and non–small-cell lung cancer) (46). The role of HER-2–targeted treatment has been confirmed in the above-mentioned tumor types, especially for those with high HER-2 expression (47, 48). In metastatic GC, approximately 6%–32% of patients are detected as HER-2–positive via an immunohistochemistry (IHC) score of 3+ or an IHC score of 2+ in combination with fluorescence *in situ* hybridization (FISH) positivity, which is significantly correlated with prognosis (46, 49, 50). Regarding HER-2 mutation, it commonly occurs in bladder cancer and cholangiocarcinoma (51).

#### 2.2.1 The mechanism of anti–HER-2 drugs

The HER-2 protein is composed of three distinct domains: extracellular domain (ECD), transmembrane domain (TMD), and intracellular domain (ICD) containing tyrosine protein kinase (RTK) activity (52–54). The ECD also includes two receptor-L domains (I and III) and two cysteine-rich domains (II and IV). The diverse drugs targeting HER-2 vary, relying on various HER-2 domains (14, 44, 55).

At present, HER-2–directed agents mainly encompass monoclonal antibodies, small-molecule tyrosinase inhibitors (TKIs), and antibody-conjugated drugs (ADCs) (51, 56). Taken trastuzumab and pertuzumab as the examples, trastuzumab frequently binds to domain IV in ECD, whereas pertuzumab predominantly binds to domain II (14, 57). Both of them are known to regulate tumor cell proliferation, metastasis, and vascularization, countering HER-2’s function. Meanwhile, the TKIs act on the ATP-binding site of the tyrosine kinase region within the intracellular region of HER-2 protein, in order to prevent the formation of HER-family dimers and to inhibit kinase phosphorylation, thereby blocking the activation of downstream signaling cascades (57). Moreover, the ADCs are engineered to link antibodies with cytotoxic agents, further delivering those drugs specifically to tumor cells through the antigen-antibody interaction while minimizing exposure to normal tissues (56). Novel antibodies such as Zanidatamab (ZW25) and KN026 are bispecific antibodies targeting HER-2, which can simultaneously bind to two distinct, non-overlapping epitopes on HER-2: the ECD domain IV (the targeting-site of trastuzumab as mentioned above) and ECD domain II (the targeting site of pertuzumab as mentioned above), in turn, to exert the dual anti-tumor effect (14, 58, 59).

#### 2.2.2 Therapy targeting HER-2 in GC

In 2010, the great success of ToGA trial established the new standard therapy of trastuzumab in the first-line treatment in patients with metastatic HER-2–postive GC (60). Compared to the chemotherapy group alone, the median overall survival (mOS) of trastuzumab combined with chemotherapy was longer (13.8 months vs. 11.1 months, HR = 0.74, P < 0.01), and the median progression-free survival (mPFS) was also prolonged (6.7 months vs. 5.5 months, HR = 0.71, P < 0.01). Moreover, the objective response rate (ORR) (47.3% vs. 34.5%) and the disease control rate (DCR) (78.9% vs. 69.3%) were respectively greater. Subgroup analysis indicated that patients with HER-2 (2+) and FISH-positive or HER-2 (3+) could benefit more from trastuzumab, with extended mOS (almost reaching 16 months). Then, in 2016 and in 2018, Hecht et al. and Tabernero et al., respectively, designed a phase III, large-arm clinical trial aiming to explore the efficacy of HER-2 blockades based on different chemotherapy regimens in GC. Unfortunately, these results have been both disappointing. In the LOGIC trial, the scholars failed to prove the efficacy of lapatinib (TKI dual-targeting EGFR and HER-2) as the first-line choice (61). The JACOB study enrolled 780 volunteers and compared triple-combination regimen (chemotherapy plus trastuzumab and pertuzumab) with double-combination regimen (chemotherapy plus trastuzumab) (62). The mOS was 18.1 months vs. 14.2 months (HR = 0.85), without a remarkable improvement.

Similarly, lapatinib in second-line therapy still failed to reveal its efficacy according to the TyTAN trial, which mainly focused on patients with HER-2 amplification (63). To overcome the acquired resistance of HER-2–directed agents, which mainly attributed to the absence of phosphatase and tensin homolog (PTEN), PI3KCA mutation, etc., novel HER-2–composed ADCs are then developed (64, 65). In 2017, a randomized, open-label and phase II/III clinical trial (named as GATSBY) referred an ADC drug (trastuzumab-emtansine, T-DM1) also showed no superiority in the mOS (ADC vs. chemotherapy, 7.9 months vs. 8.6 months, HR = 1.15, P = 0.86) and the mPFS (2.7 months vs. 2.9 months, HR = 1.13, P = 0.31) (66). Similarly, another study, DESTINY-Gastric01, compared the efficacy of trastuzumab-deruxtecan (T-DXd/DS-8201a) with irinotecan/paclitaxel for those undergoing the second-line therapy (67). The ORR in those receiving trastuzumab-deruxtecan was remarkably higher than those under irinotecan or paclitaxel treatment (51% vs. 14%). More intriguingly, in two cohorts that mainly focused on those with HER-2–low (defined as IHC score of 2+ or IHC score of 1+ but FISH-negative), the ORR in trastuzumab-deruxtecan group and the control group were 26.3% and 9.5%, respectively. Subsequently, trastuzumab-deruxtecan has been approved as the current second-line choice in GC, as well as ramucirumab plus paclitaxel. Furthermore, several ongoing clinical trials are presently under evaluation for the use of other ADCs, such as tucatinib, margetuximab, and zanidatamab (68).

### 2.3 The role of PI3K/AKT/mTOR signaling pathway

The phosphatidylinositol-3-kinase (PI3K), a member of the lipid kinase family, is categorized into Class I, Class II, and Class III (29, 69). The Class I PI3K consisting of class IA and class IB subtype is involved in the cell-growth signal transmission (69–71). AKT is the key downstream effector of PI3K, with three subtypes including AKT1, AKT2, and AKT3 (43, 72, 73). After triggering by upstream tyrosine kinase receptors (RTKs), the Class I PI3K is activated and subsequently phosphorylates phosphatidylinositol-4,5-bisphosphate (PIP2) to phosphatidylinositol-3,4,5-trisphosphonate (PIP3). Then, PIP3 interacts with the Pleckstrin Homolgy (PH) domain of AKT, like a second messenger, to further transport AKT from the cytoplasm to the membrane (74). Thus, the conformational change occurs. The Ser473 and Thr308 threonine residues of AKT are activated by phosphorylation of phosphoinositol-dependent kinase 1 (PDK1) and mammalian target of rapamycin complex 2 (mTORC2), respectively (75–77). Phosphorylated AKT (p-AKT) could directly activate the mTOR signaling pathway or indirectly activate the mTOR signaling pathway by inhibiting tuberous sclerosis complex 1/2 (TSC1/2) (77, 78). In addition, activated mTORC1 participates in downstream-protein translation, cell growth, and proliferation via translation initiation factor (4E-BP1) and p70 ribosomal protein kinase S6 (p70S6K) (77, 79).

It is reported that the mutation in exon 9 of the *PIK3CA* gene presumably predict poor prognosis in patients with EBV-associated GC (74, 80, 81). Another in-depth study indicated that mutations in exon 9 of *PIK3CA* are closely related with poor prognosis in GC compared to mutations in exon 20 (82, 83). In addition, a lower 5-year survival rate was observed in those patients with MSI GC with *PIK3CA* mutation than those without the above mutation. *PIK3CA* amplification, accompanied an elevation in AKT and its phosphorylation levels, eventually promotes invasion and lymph node metastasis in GC.

### 2.4 The role of hepatocyte growth factor/mesenchymal epidermal transition factor signaling pathway

c-MET, a transmembrane tyrosine kinase that expressed on epithelial and endothelial cells, is encoded by *MET* gene (84). The hepatocyte growth factor (HGF) is the specific known high-affinity ligand for c-MET and belongs to the family of plasminogen associated growth factors (PRGF-1) (85, 86). When HGF binds with c-MET, c-MET dimerization forms to induce self-phosphorylation of residue Y1234 and Y1235 (85, 87). Its downstream molecules, such as growth factor receptor binding protein 2 (GRB2), GRB2-related binding protein (GAB1), Src homologous region 2 protein tyrosine phosphatase 2 (SHP2), and PI3K, are recruited and are then amplified through a phosphorylation reaction cascade to activate PI3K/AKT and MAPK axis. The above crosstalk jointly contributes to tumor invasion and metastasis (88).

The prognosis of GC driven by *MET* gene is generally poor (89–91). Abnormal c-MET signals have been reported in various tumor studies, mainly including *MET exon 14* mutation, MET amplification, and MET protein overexpression (92, 93). However, detection measurements via FISH, droplet-based digital PCR (ddPCR), or next-generation sequencing (NGS) would cause discrepancy. Y1003 and c-CblE3 ubiquitin ligase binding sites (located in *MET exon 14*) are missing, resulting in delayed ubiquitination and sustained activation of c-MET as well. Moreover, it is estimated that there are approximately 4%–6% of patients with MET-amplified GC (92). Furthermore, overexpression of c-MET in GC is positively correlated with higher risk of distant metastasis (like peritoneum, liver, and lung), especially carcinomatous lymphangitis (94, 95). Inhibitors targeting MET are also extensively studied in GC. For instance, a single-arm, multi-cohort, multi-center, open-label, and phase II clinical study aimed at evaluating the efficacy and safety of savolitinib monotherapy in advanced/metastatic GC with MET amplification (the VIKTORY trial, NCT04923932) (96). Twenty patients were totally enrolled. Notably, the ORR in 16 high *MET* gene copy reached 50%, which indicated the value of c-MET inhibitor in GC. Another anti-MET drug (onartuzumab) failed to improve efficacy in the phase III trial (METGastric) (97). Similarly, the RILOMET-1 and RILOMET-2 study emphasizing rilotumumab in GC/GEJC with MET(+) were both terminated attributing to the increasing death of the rilotumumab arm (98). Altogether, targeting c-MET is promising but challenging.

### 2.5 The role of fibroblast growth factor receptor signaling pathway

Fibroblast growth factor receptor (FGFR) bound with fibroblast growth factors (FGFs) is widely involved in tumor invasion, differentiation, and angiogenesis (99–101). In GC, the common abnormalities mainly consist of *FGFR1* gene alteration, *FGFR2* amplification, and *FGFR3* rearrangement (102). After integrating with FGF, the phosphorylation-induced FGFR activation occurs, followed by the activation of MAPK and PI3K/AKT pathway (102). It was reported that approximately 4.1% of GC cases were detected as amplification in *FGFR2* (102–104). The existing data have emphasized the potential of FGFR as a biomarker. The FGFR2b-targeted agent, bemarituzumab (a humanized IgG1 monoclonal antibody), has been confirmed its potential in a phase II FIGHT trial when used as the first-line treatment plus mFOLFOX standard chemotherapy (5-FU + leucovorin + oxaliplatin) in GC (105). Compared with the placebo with mFOLFOX, the addition of bemarituzumab led to a higher ORR (47% vs. 33%) and a longer PFS (9.5 months vs. 7.4 months). More importantly, for those with FGFR2b-positive receiving bemarituzumab, an obvious OS benefit was observed (25.4 months vs. 11.1 months, P < 0.001). A more-sample and phase III clinical trial about bemarituzumab plus ICIs is currently being investigated. In 2017, Van Cutsem et al. that AZD4547 (a selective *FGFR-1*, *FGFR-2*, or *FGFR-3* TKI) monotherapy failed to prolong the mPFS versus paclitaxel (1.77 months vs. 2.12 months, P = 0.9581) (106). Other selective *FGFR* inhibitors (such as derazantinib and futibatinib) are also ongoing (107, 108).

### 2.6 The role of VEGF/VEGFR signaling pathway

During the growth of tumors, new blood vessels are warranted. Angiogenesis driven by high-level VEGF is common in the solid tumors, as well as in GC (12, 109). VEGFR2, the main receptor for VEGF-induced signal transduction in endothelial cells, self-phosphorylates and is activated when binding with VEGF (110–112). The phosphorylation of VEGFR2 at Tyr1212 provides a docking site for GRB2 binding, whereas phosphorylation at Tyr1175 leads to the binding with p85 subunit of PI3K and PLCγ (113). VEGFR2 is activated during angiogenesis and can be transduced through multiple downstream pathways, including AKT, p38, and ERK 1/2, and is involved in regulating cell proliferation and migration (111, 112). The activation of hypoxic pathways can also participate in tumor angiogenesis via the upregulation of VEGF. The core of the hypoxic pathway is the hypoxia-inducible factor-1 (HIF-1) complex, which consists of two subunits (HIF-1α and HIF-1β) (114, 115). Activated by the proline hydroxylase domain (PHD), such as PHD-1, PHD-2, and PHD-3, HIF-1α hydroxylation occurs, which is then combined with VHL E3 ligase and degraded through the ubiquitination proteasome pathway under normoxic conditions (114). However, the lack of oxygen would upregulate HIF-1α and subsequently activate the downstream (including the VEGF) to promote angiogenesis (116). It was reported that the HIF-1α expression in GC could predict poor prognosis (117). When blocking angiogenesis via anti-VEGF or anti-VEGFR therapy, the secretion of pro-angiogenic cytokines is correspondingly decreased. **Table 2** showed that the clinical trials involved anti-angiogenesis agents in advanced GC.

**Table 2:** Current clinical trials about anti-angiogenesis agents in unresectable or metastatic gastric cancer.

Clinical trial	Regimen	Line	Phase	Number	OS (months)	PFS (months)
RAINBOW (118)	Paclitaxel + ramucirumab vs. ramucirumab	2L	III	668	9.6 vs. 7.4 (HR = 0.81)	10.0 vs. 8.1 (HR = 0.64)
RAINBOW-Asia (118)	Paclitaxel + ramucirumab vs. ramucirumab	2L	III	392	9.03 vs. 8.08 (HR = 0.963)	4.17 vs. 3.15 (HR = 0.765)
REGARD (119)	Ramucirumab vs. placebo	2L	III	335	5.2 vs. 3.8 (HR = 0.78)	2.1 vs. 1.3 (HR = 0.48)
INTEGRATE (120)	Regorafenib vs. placebo	1/2L	II	592	NA	2.6 vs. 0.9
LSK-ANGEL (121)	Apatinib + BSC vs. placebo + BSC	2/3L	III	460	5.78 vs. 5.13 (HR = 0.93)	2.83 vs. 1.77 (HR = 0.57)
Li et al. (122)	Apatinib vs. placebo	3/4L	III	273	6.5 vs. 4.7 (HR = 0.71)	2.6 vs. 1.8 (HR = 0.44)
FRUTIGA (123) (NCT03223376)	Fruquintinib + paclitaxel vs. paclitaxel	2L	III	699	NA	5.6 vs. 2.7 (HR = 0.57)

BSC, best support care; 1L, first line; 2L, second line; 3L, third line; PFS, progression-free survival; OS, overall survival; HR, hazard ratio; NA, not available.

According to the results from the REGARD and RAINBOW trials, the widely recognized agent, ramucirumab (a recombinant VEGFR-2–directed monoclonal antibody), has been approved as the second-line application in GC (118, 119). Intriguingly, ramucirumab monotherapy indicated improvement in mOS compared with placebo (the REGARD trial) (119). In addition, in the RAINBOW trial, the setting of ramucirumab combined with paclitaxel had a prolonged survival than the paclitaxel arm (9.6 months vs. 7.4 months). Another oral and small-molecule TKI, apatinib, selectively inhibits VEGFR-2. A randomized phase III trial in China revealed that apatinib prolonged the mOS versus placebo in the third-line setting and beyond (6.5 months vs. 4.7 months) (122). However, the adverse events induced by apatinib restrict its application in clinic. Subsequently, the investigators designed a double-blind phase III study (FRUTIGA, NCT03223376) aiming to compare the efficacy of fruquintinib plus paclitaxel versus paclitaxel monotherapy as the second-line setting in advanced GC/GEJC (124). According to the preliminary results, when coupled with paclitaxel, fruquintinib significantly improved the PFS, the ORR, and the DCR. However, a similar benefit failed to be observed in overall survival (OS). The final data from FRUTIGA is still under analysis.

As is described below, anti-angiogenic drugs can stimulate the immune system so that the addition of ICIs could have synergistic anti-tumor effect and overcome resistance. In an open-label, phase Ib REGONIVO trial, the scholars claimed that the ORR of those patients with GC who received the combination of regorafenib and nivolumab therapy reached 44%, and the OS of whom was 5.6 months (125). In 2024, Yongqian Shu et al. designed the first phase I clinical trial (the SPACE) that explored the efficacy of apatinib plus camrelizumab and chemotherapy as the first-line treatment in unresectable or metastatic GC (126). Among the 34 patients, the ORR reached 76.5%. Moreover, 10 patients underwent curative resection. The researchers also observed that patients with a higher percentage of tertiary lymphatic structure and a higher baseline infiltration of CD3^+^ or Foxp3^+^cell density had a longer OS. Taken together, despite efforts made in anti–angiogenic-related trials, no well-defined biomarkers have been currently established to guide angiogenesis blockades selection.

### 2.7 The role of Claudin 18.2 (CLDN 18.2)

Claudin proteins (CLDNs) typically participate in intercellular tight-junction (127). However, malignant tumor could disrupt this adhesion, therefore exposing CLDNs epitope on the surface of tumor cells (128, 129). The CLDN 18.2 encoded by *Claudin 18.2* gene could be particularly detected in the gastric mucosa (129). However, aberrant upregulation of the CLDN 18.2 (approximately 60%–80%) was found in GC, which has been a novel and promising therapeutic target based on the existing data as the later-line selection (23, 127, 130).

CLDN 18.2–targeted antibody is emerging as a promising anti-tumor agent via antibody-dependent cytotoxicity (ADCC) (130). Zolbetuximab (IMAB362, Claudixmab) is a human-mouse chimeric Immunoglobulin G2 (IgG2) monoclonal antibody targeting claudin 18.2, which specifically bind to claudin 18.2 and then lead to ADCC and apoptosis. In 2021, Sahin et al. initiated the FAST trial, a randomized and phase II trial, which included a total of 334 patients with advanced GC/GEJC with CLDN 18.2–positive and compared the efficacy of zolbetuximab plus chemotherapy (epirubicin + oxaliplatin + capecitabine, EOX regimen) with single EOX as the first-line treatment (131). The results showed that the mPFS and the mOS were both prolonged in the zolbetuximab plus EOX group (7.5 months vs. 5.3 months and 16.5 months vs. 8.9 months, respectively). Of note, the sub-analysis indicated that those with CLDN 18.2 level ≥70% of tumor cells could benefit more from zolbetuximab. To further explore its value in the first-line therapy in GC, two phase III large-scale clinical trial emerged, named as the SPOTLIGHT (NCT03504397) and the GLOW (NCT03653507). In the SPOTLIGHT trial, participants were randomly divided into the zobezumab + mFOLFOX group (n = 283) or the placebo + mFOLFOX6 group (n = 282) in a 1:1 ratio (132). Compared with placebo, the adding of zolbetuximab prolonged the mPFS (10.61 months vs. 8.67 months, P = 0.0066) and the mOS (18.23 months vs. 15.54 months, P = 0.0053) as well. In addition, the safety was tolerable and manageable. As for the GLOW, this randomized, double-blind, placebo-controlled research was designed to evaluate the potential of zolbetuximab plus cisplatin + capecitabine (CAPOX) in patients with unresectable/metastatic GC/GEJC with CLDN 18.2(+) and HER-2(−) (133). Compared with placebo, the adding of zolbetuximab revealed significant benefits in the mPFS, with median PFS of 8.21 months vs. 6.8 months (P = 0.0007). Moreover the 1-year PFS rate in the zolbetuximab + CAPOX arm and the placebo + CAPOX arm was 35% and 19%, respectively. Similarly, the mOS in the zolbetuximab + CAPOX group was obviously longer than that in the placebo + CAPOX group (14.39 months vs. 12.16 months, P = 0.0118). In the phase IIa trial (MONO), 54 patients with GC/GEJC were enrolled to receive single zolbetuximab as the later-line treatment (134). Among them, 10 patients reached disease remission. Another CLDN 18.2–directed ADC, ATG-022, has been approved as orphan drug in GC by Food and Drug Administration (FDA). The preclinical data demonstrated that ATG-022 exhibited a strong *in vivo* anti-tumor effect in GC patient-derived tumor xenograft model with high-expression CLDN 18.2 (135). Consequently, the CLINCH trial (NCT05718895) related with ATG-022 is ongoing. It is worth noting that the potential damage of normal gastric mucosal induced by CLDN 18.2–directed antibody or ADCs should also be taken seriously into consideration.

In addition, several phase I trials found that CLDN 18.2–specific CAR-T therapy brought an encouraging tumor regression in patients with GC (115, 136–139). For example, in 2021, the scholars represented that CT041 predominantly improved the tumor control rate in GC/GEJC as the third-line treatment. The ORR was 61.1%, and the DCR was 83.3%, both of which were significantly higher than chemo-regimen or ICIs. Then, professor Lin Shen et al. recruited 28 patients with CLDN 18.2(+) GC/GEJC who have previously failed at least second-line treatment (NCT03874897) (115). The 6-month OS rate reached 81.2%. More importantly, the ORR and the DCR were 57.1% and 75.0%, respectively. The barriers, such as the tumor heterogeneity, safety managements and high-cost, remain challenging.

## 3 The applications of immune checkpoint inhibitors in gastric cancer

### 3.1 The immune checkpoint signaling pathway in gastric cancer

Programmed cell death 1 (PD-1) and PD-L1 are two well-recognized immune checkpoints across various tumor types (140). As a whole, PD-1 is typically found on the surface of activated T cells, B cells, dendritic cells (DCs), and natural killer (NK) cells. It interacts with PD-L1/programmed cell death ligand 2 (PD-L2) on tumor cells, contributing to the formation of an immunosuppressive microenvironment (141). Likewise, cytotoxic T lymphocyte antigen 4 (CTLA-4) is another vital immune checkpoint, which engages with B7 on antigen-presenting cells to collectively promote GC immune escape (142). In addition, lymphocyte-activation gene 3 (LAG-3) is not expressed on naive T cells. Sustained antigen stimulation triggers LAG-3 expression on both CD4^+^ and CD8^+^ T cells, which helps prevent autoimmune damage, gradually followed by T-cell dysfunction (143, 144). T-cell immunoglobulin and mucin-domain containing-3 (TIM-3) interacted with galactin-9 or galactin-3 and T-cell immunoreceptor with Ig and ITIM domains (TIGIT) binding to CD155 also contribute to immune escape (145–147) (**Figure 1**). Consequently, blocking PD-1/PD-L1 or CTLA-4 pathways can restore and reactivate T cells, thereby inducing an anti-tumor effect (148, 149).

### 3.2 First-line treatment

#### 3.2.1 Pembrolizumab

##### 3.2.1.1 Pembrolizumab in HER-2–negative GC

To explore the value of pembrolizumab in patients with unresectable or metastatic GC/GEJC with HER-2(−), the researchers firstly investigated a multi-center, randomized, partial-blind, and phase III trial (KEYNOTE 062, NCT02494583) (150). The participants were randomized into three arms (the pembrolizumab monotherapy arm, the pembrolizumab plus CAPOX/FOLFOX arm, and the placebo plus chemotherapy arm). Interestingly, for those with PD-L1 combined positive score (CPS) ≥1, pembrolizumab showed non-inferiority to standard chemotherapy (10.6 months vs. 11.1 months, HR = 0.91) (151). Moreover, subsequent analysis demonstrated that pembrolizumab significantly prolonged the mOS than chemotherapy alone among those with CPS ≥10 (17.4 months vs. 10.8 months, HR = 0.69). Of note, results from the sub-analysis data found that the PFS in the pembrolizumab group failed to be prolonged compared with the chemotherapy group (2.0 months vs. 6.4 months referring to the population with CPS ≥1. Taking the long-term survival benefit into account, pembrolizumab presumably contributes more than chemotherapy. Just on the basis of KEYNOTE 062, another placebo-controlled and phase III trial (KEYNOTE 859, NCT03221426) adjusted the chemo-regimen [fluorouracil + cisplatin (FP) or CAPOX] and then evaluated the efficacy of pembrolizumab plus chemotherapy versus chemotherapy alone when as the first-line treatment (152, 153). Overall, a slight improvement was observed in the OS (12.9 months vs. 11.5 months, HR = 0.78) and the PFS (6.9 months vs. 5.6 months, HR = 0.76). In addition, the further sub-analysis showed that the addition of pembrolizumab consistently gained benefits in various subgroups.

In 2023, the LEAP-015 (NCT04662710), a randomized, open-label, two-part, and phase III clinical trial, was designed by Kohei Shitara et al. (154). According to the data from the run-in phase of the LEAP-015 (part I), the preliminary anti-tumor effect was observed in the pembrolizumab + lenvatinib (a multi-receptor TKI) + chemotherapy group (ORR, 73%; DCR, 93%). In addition, the safety is controllable. Part II is recruiting patients with locally advanced/metastatic GC/GEJC with HER-2(−) who were not previously treated to investigate the efficacy of the pembrolizumab + lenvatinib + chemotherapy regimen versus chemotherapy alone.

##### 3.2.1.2 Pembrolizumab in HER-2–positive GC

The KEYNOTE 811 trial (NCT03615326) mainly enrolled 698 patients with advanced GC with HER-2(+), aiming to elucidate the potential of pembrolizumab plus trastuzumab and chemotherapy (XELOX or PF) (155). In detail, from the third mid-term analysis, the mPFS in the pembrolizumab + trastuzumab + chemotherapy group and in the placebo + trastuzumab + chemotherapy group was 10.0 months vs. 8.1 months (HR = 0.73), especially in those with PD-L1 CPS ≥1. Adding pembrolizumab also resulted in a higher ORR (74.4% vs. 51.9%). However, the mOS was 20.0 months vs. 16.8 months (HR = 0.84), respectively, which did not reach statistically significant difference. These encouraging results have prompted rapid approval of pembrolizumab coupled with trastuzumab and chemotherapy as the first-line setting in HER-2–postive unresectable or metastatic GC.

#### 3.2.2 Nivolumab

Similar to the KEYNOTE 062, Kang et al. initiated a multi-center, double-blind, placebo-controlled, phase II/III study (ATTRACTION-04, NCT02746796), in which the aim was to explore the safety and effect of nivolumab based on SOX (S-1 + oxaliplatin) or CAPOX in the first-line setting among 40 patients with advanced GC/GEJC with HER-2–negative (156). However, the ATTRACTION-04 mainly highlighted on the Asian population. Furthermore, it did not consider the PD-L1 expression as enrollment standard. The regimen of nivolumab plus chemotherapy led to a longer PFS than chemotherapy alone (10.45 months vs. 8.34 months, P = 0.0007), whereby no obvious OS improvement in two groups (17.45 months vs. 17.15 months, P = 0.26). Another large-sample phase III trial (CheckMate-649, NCT02872116) was the largest-scale research in GC immunotherapy to date (157). Approximately 1,581 participants were recruited. The main endpoint was the mOS and the mPFS in those with PD-L1 CPS ≥5. Compared with chemotherapy alone, the addition of nivolumab to chemotherapy obviously prolonged mOS (14.4 months vs. 11.1 months, P < 0.0001) among the CPS ≥5 population, as well as the mPFS (7.7 months vs. 6.05 months, P < 0.0001). Currently, at the American Society of Clinical Oncology (ASCO)–GI in 2024, the scholars updated the 4-year follow-up results of Chinese patients. The 4-year OS rate among the entire population has reached 21%. As for the participants in China, this objective was higher, nearly reaching 25%. In the population harboring PD-L1 CPS ≥5, the mPFS in the nivolumab + chemotherapy arm almost doubled that in the chemotherapy arm (8.5 months vs. 4.3 months, respectively).

#### 3.2.3 Sintilimab

The emergence of the ORIENT-16 trial provides a novel combination approach based on sintilimab plus oxaliplatin–based chemo-regimen in advanced GC/GEJC when regarded as the first-line therapy (158, 159). A total of 650 patients were included and then were randomly assigned into the sintilimab + CAPOX group or the placebo + CAPOX group. Final analysis results indicated that the mOS in the sintilimab-treated arm were extended by 2.9 months targeting the overall population (15.2 months vs. 12.3 months, P < 0.0001). A similar improvement of the mOS was equally observed in the population with PD-L1 CPS ≥5, 19.2 vs. 12.9 months (HR = 0.66, P < 0.0001). Moreover, the benefit was consistent across subgroup analysis. The frequent treatment-related adverse events (AEs) were decreased platelet and neutrophil count.

#### 3.2.4 Tislelizumab

Tislelizumab (BGB-A317) is another anti–PD-1 agent and is under further evaluation in the RATIONALE-305 trial (160). Also, 997 patients worldwide who have not received systematic treatment joined in the phase III trial. In the ITT (defined as intention-to-treat) population and PD-L1–positive population (defined as tumor area positivity score ≥5%) treated by tislelizumab, the 2-year durable rate of response (DOR) nearly reached 30% and 40%, respectively. By comparison, that of the chemotherapy group was less than 20%. Furthermore, the 2-year PFS rate of the tislelizumab arm in the ITT population and the PD-L1(+) population was 17.6% and 22.3%, whereas that of the chemo arm was 9.1% and 8.7%. Similarly, these data claimed a durable response driven by tislelizumab (160).

#### 3.2.5 Sugemalimab

Sugemalimab (CS1001) is a PD-L1–targeted IgG4 monoclonal antibody. A randomized, double-blind, phase III clinical research (GEMSTONE-303) aimed to evaluate the efficacy of sugemalimab + CAPOX versus placebo + CAPOX in first-line treatment of advanced GC/GEJC adenocarcinoma with PD-L1 CPS ≥5 (161). The PFS and the OS both met the endpoint. Compared with CAPOX alone, the addition of sugemalimab improved the PFS (7.62 months vs. 6.08 months, P < 0.0001) and the OS (15.64 months vs. 12.45 months, P = 0.0060). Notably, in the population with PD-L1 CPS ≥10, a more obvious benefit in the PFS and the OS was observed. The GEMSTONE-303 firstly and accurately screened the population with PD-L1 CPS ≥5 in advanced GC, and, in turn, the viewpoint that GC treatments should be precisely selected has been further clarified.

#### 3.2.6 Avelumab

The value of ICIs maintenance treatment after induction chemotherapy was described in the JAVELIN Gastric 100 (NCT02625610), which failed to show superiority in OS (162). Taking the 24-month OS rate for example, that in the avelumab maintenance arm and in the continued chemotherapy arm was 22.1% and 15.5% (P = 0.1779), respectively.

According to the results from the trials above, anti–PD-1/anti–PD-L1 drugs based on standard chemotherapy have been confirmed its vital role in first-line treatment of HER-2(−) advanced GC/GEJC harboring PD-L1–positive (CPS ≥5, or even CPS ≥10). However, it remains controversial whether those with low-expression PD-L1 (or unknown expression) could benefit from ICIs. As for those with HER-2(+), combining with HER-2–directed therapy is also recommended. Another question is the feasibility of dual-ICI regimen in GC when used as the first-line setting (such as nivolumab plus ipilimumab), awaiting further investigation in the future.

### 3.3 Second-line treatment

#### 3.3.1 ICI monotherapy

ICIs have challenged the existing role of standard chemotherapy as the first-line choice. To further assess its efficacy in the second-line treatment, the KEYNOTE 061 trial (NCT02370498) recruited 592 patients with GC/GEJC after previous therapy (163). In detail, pembrolizumab monotherapy did not prolong the mOS compared to paclitaxel (9.1 months vs. 8.3 months, P = 0.042). Even in terms of the PFS, that of the pembrolizumab arm and the paclitaxel arm was 1.5 months and 4.1 months, respectively. As a result, pembrolizumab alone as the second-line treatment declared failure. However, in-depth analysis from the sub-group results indicated that an improved OS was observed in those PD-L1(+) population, regardless of CPS ≥1, CPS ≥5, or CPS ≥10 (164). It is consistent with the previous hypothesis that ICI utilization needs to be more precise based on the PD-L1 level.

#### 3.3.2 ICIs plus anti-angiogenesis agents

Just as previously introduced in preclinical research studies, fruquintinib targeting VEGFR axis could enhance the infiltration of cytotoxic T cells and reduce PD-1–positive CD8^+^ cells. Simultaneously, regulating Tumor-associated macrophages (TAMs) and promoting M_1_ macrophage polarization both trigger fruquintinib to synergistically kill tumor cells with ICIs. The feasibility of combination of fruquintinib plus ICIs has been confirmed in several preliminary small-sample trials, which provides an option of chemo-free when progressed after the failure from first-line chemotherapy. In the ASCO-GI 2024 meeting, the investigators published the updated data of fruquintinib plus sintilimab in 14 patients with advanced GC/GEJC after failure of platinum-based regimen (123). The ORR was 33.3%, and the DCR was 66.7%. Only one patient experienced grade 3/4 treatment-related adverse events (TRAEs). Preliminarily, fruquintinib plus sintilimab is efficient and tolerable in safety.

### 3.4 Later-line treatment (third line and beyond)

In clinic, when the patients progressed after 1L and 2L treatments, the proportion of patients who can receive the later-line therapy significantly decreases as a result of the poor performance status. Overall, chemotherapy regimens including docetaxel or irinotecan in 3L treatments have limited survival benefits, with a mOS of 5.3–5.8 months and a mPFS of approximately 2–3 months. According to the ATTRACTION-2 (NCT02267343) and the KEYNOTE 059 trial (NCT02335411), pembrolizumab and nivolumab alone have been both approved as a third-line option in advanced GC/GEJC (165, 166). In the KEYNOTE 059, 259 patients with advanced GC who previously received treatments planned to underwent monotherapy with pembrolizumab (166). The final data showed that the PFS and the OS were 2.0 months and 5.6 months, respectively. In addition, the ORR was 11.6%. Of note, cohort 3 aimed to those harboring PD-L1(+) (defined as CPS ≥1), and the ORR of that reached 15.5%. The ATTRACTION-2, a randomized, multi-center, placebo-control, and phase III clinical trial, recruited 493 patients with GC/GEJC (165). Despite PD-L1 expression, the nivolumab arm achieved a longer OS than placebo (5.32 months vs. 4.14 months, P < 0.0001), accompanied by manageable AEs. Another phase III JAVELIN Gastric 300 trial (NCT02625623) failed to confirm the efficacy of avelumab in the third-line setting (167).

Although ICIs are feasible in the third-line treatment for patients with metastatic GC, the benefits are still restricted. How to choose the best-responder and seek the potential beneficiary is crucial in the future.

## 4 Potential molecular biomarkers in target-based and ICI-based treatment for GC

**Figure 2:**
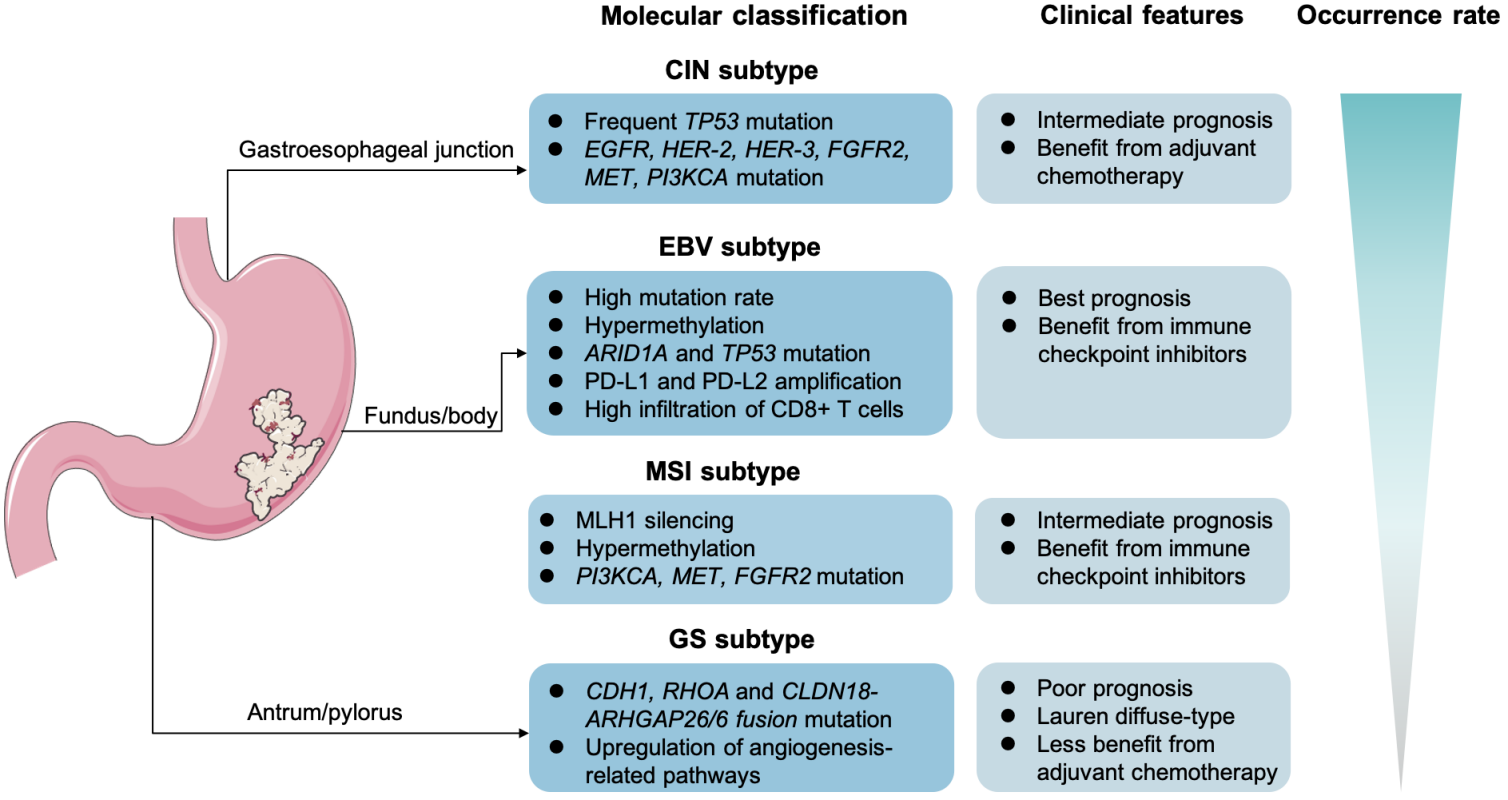
Molecular classification and clinical features in gastric cancer. The Epstein–Barr virus infection–related type (EBV-positive), the microsatellite unstable type (MSI), the chromosomal unstable type (CIN), and the genomically stable type (GS).

In recent years, the rapid advancements in genomics and innovative therapeutic strategies, including targeted therapy and ICIs, have significantly shifted the landscape of GC treatment toward precision and personalized medicine. The continuous advancements in molecular detection methods, such as NGS, whole-exon sequencing (WES), and the liquid biopsy, unveil novel targets to further facilitate medication selection and efficacy prediction (168). Stratification and molecular classification at initial diagnosis are relatively essential in GC management. The Lauren classification defined GC as diffuse type, intestinal type, and mixed type. In detail, the intestinal GC is mostly seen in the elderly and men, which is often considered to be secondary to chronic atrophic gastritis. Yet, the diffuse-type GC cells generally lack cell adhesion and fail to form glandular ducts, with *CDH1* gene germline mutations. Compared with the intestinal GC, the diffuse type is more frequent in young women. Another point is that the diffuse type is prone to lymph node metastasis and distant metastasis. Notably, The Cancer Genome Atlas recommended to clarify patients with GC into four subtypes, consisting of the Epstein–Barr virus infection–related type (EBV-positive), the microsatellite unstable type (MSI), the chromosomal unstable type (CIN), and the genomically stable type (GS) (4, 169) (**Figure 2**). The CIN type usually tends to present as intestinal phenotype, whereas the GS type mostly presents as diffuse phenotype. Then, in 2015, the Asian Cancer Research Group proposed another clarification system, mainly highlighting on the microsatellite status and *TP53* activation. Specifically, the researchers categorized GC into the MSI, the microsatellite stable (MSS; or defined as EMT), the MSS and *TP53*(+), or the MSS but *TP53* deficiency subtype. According to molecular features in GC, the different clarification could predict clinical outcomes. For instance, it is reported that the GS subtypes in GC is often associated with a poorer prognosis and lower sensitivity to chemotherapy (170). In contrast, the MSS/EMT subtype, frequently marked by the loss of *CDH1*, tends to be more prevalent in younger patients (171). However, those with MSI-high or EBV-positive are generally considered to benefit more from immunotherapy.

### 4.1 HER-2 amplification

HER-2 amplification is of great significance in precision medicine. Based on the analysis from the ToGA, the LOGIC, and the JACOB trial, it is obvious that patients with HER-2–positive advanced GC can benefit from anti–HER-2 therapy (60–62). Moreover, the KEYNOTE 811 study also indicates that those patients with GC with HER-2 amplification would be suitable for ICIs plus HER-2–targeted therapy (155). Therefore, HER-2 status can further predict the therapeutic response and survival benefits of advanced GC. According to HER-2 detection methods, IHC is common. In addition, IHC 0/1+ or IHC 2+ with no amplification of FISH can be directly judged as HER-2–negative. IHC 3+ or IHC 2+ and FISH amplification are determined as HER-2–positive. For blood samples, the copy number of *HER-2* gene somatic cells based on ctDNA targeted sequencing in blood is highly consistent with FISH data. For patients who cannot receive biopsy, liquid biopsy toward HER-2 is recommended. More importantly, precise screening of HER-2–positive GC populations urges a combination of multiple methods in the future.

### 4.2 Microsatellite status

The major function of mismatch repair proteins (MMR) is to correct and to fix the errors during DNA replication. If deficiency or loss occurs in MMR genes (including *MLH1*, *MSH2*, *MSH6*, and *PMS2*), then we defined it as dMMR, which is generally equivalent to MSI-high (MSI-H). Hence, dMMR results in a continuous accumulation of mutation-induced errors, then triggering malignant transformation. Increasing evidence suggested that the MSI-H tumors show better response to immunotherapy (GC included) (20, 172). One explanation is attributed to the increase tumor-specific neoantigens and tumor-infiltrating lymphocytes (TILs) (173, 174). As discussed earlier, the KEYNOTE 061, KEYNOTE 062, CheckMate-649, and JAVELIN gastric 100 trials demonstrated that patients with GC with dMMR/MSI-H had a better clinical outcome when treated with ICI monotherapy or ICIs plus chemotherapy (175). Furthermore, the subgroup analysis from the KEYNOTE 062 showed that an obvious improvement of PFS and OS was observed in the MSI-H population regardless of receiving ICI monotherapy or ICIs plus chemotherapy (151). In detail, there were 14 MSI-H participants under pembrolizumab alone. The ORR was 57.1%, and the PFS was 11.2 months. When coupled with chemo-regimen, 17 patients with dMMR were treated with pembrolizumab + chemo. Compared to the MSS arm, the ORR in the MSI-H arm was much higher (64.7%). A pan-tumor clinical trial totally enrolled 108 patients with MSI-H/dMMR (including GC/GEJC) and explored the efficacy of serplulimab (NCT03941574) (176). The ORR reached 38.2%. It is worthy to note that the 1-year OS rate and the 1-year PFS rate were 81.9% and 61.9%, respectively. Given that MSI/dMMR is a vital prediction biomarker of immunotherapy, it is recommended to routinely evaluate the MMR status for patients with GC before anti–PD-1/anti–PD-L1 treatment in clinic.

### 4.3 PD-L1 expression

The role of PD-L1 as a biomarker has been widely discussed. It seems that the relationship between PD-L1 expression and the response to immunotherapy is definite; yet, what is the appropriate and uniform cutoff value failed to reach a consensus (177). Cohort I of the KEYNOTE 059 showed that the PD-L1 CPS ≥1 group had a higher ORR than that of the CPS <1 group (15.5% vs. 6.4%) (166). The KEYNOTE 061 found that, in patients with PD-L1 CPS ≥1, ≥5, and ≥10, pembrolizumab extended OS by 0.8 months, 1.9 months, and 2.4 months compared to paclitaxel monotherapy, respectively. However, in the CheckMate-649 and ORIENT-16 trials, the cutoff value of CPS was set to 5 (158, 178). Indeed, the PD-L1 CPS is positively correlated with clinical benefits. Just based on the existing data, we cannot distinguish whether patients with CPS 1–4 will definitely not benefit from immunotherapy. Another cause for this dilemma is the diversity of measurements and interpretations, such as 22C3 pharma Dx, SP 142, and SP 263 (179, 180). Apart from CPS, several trials utilize tumor proportion score (TPS) and TAP as well (179, 181). Taking the RATIONALE-305 as an example, the investigators chose TAP to interpretate PD-L1 expression. It remains controversy over the spatiotemporal heterogeneity of PD-L1 detection and the heterogeneity of the primary metastatic lesion, as well as the heterogeneity between CPS, TPS, and TAP, all of which require further exploration.

### 4.4 EBV infection

Approximately 10% of patients with GC are diagnosed with EBV infection (EBV-associated GC, EBVaGC), and, in turn, sustained infection causes increased the infiltration of CD8^+^ T cells (such as CD8^+^ PD-1^−^ LAG-3^−^ T cells), as well as the upregulation of PD-L1 and PD-L2 (19, 182, 183). As a result, EBVaGC is thought eligible for immunotherapy and has a good prognosis (184). Moreover, low frequency of lymph nodes involvements might be another feature in EBVaGC (185). A small-sample study demonstrated that the response rate in EBV-positive GC reached 100% (n = 6) (95). Detecting EBV via EBV-encoded RNA *in situ* hybridization (EBER-ISH) becomes the gold standard (186). With the increasing demand for biomarker detection, only relying upon EBER-ISH is not enough in clinical practice. NGS panel detecting of EBV status at RNA level has emerged, including seven EBV genes (*EBER1*, *EBER2*, *EBNA1*, *LMP1*, *LMP2A/B*, *BZLF1*, and *BARF1*) (186).

### 4.5 Tumor mutation burden

Tumor mutation burden (TMB) is defined as the total number of somatic alterations detected per million bases (muts/Mb) (187). Previous evidence illustrated that TMB could serve as a biomarker independently of MSI-H and PD-L1 in immunotherapy for GC (188, 189). Typically, the TMB-high (TMB-H) status accompanies with the exposure of neoantigens and the further recognition by antigen-presenting immune cells, like DCs (187). Thus, the tumor cells are more vulnerable to anti–PD-1/anti–PD-L1 agents; that is to say, those with TMB-high in GC can benefit more from ICIs. In 2019, Professor Xu et al. reported a phase Ib/II trial (NCT02915432) in GC referring to toripalimab (190). A higher ORR was seen in the candidates with TMB-H (TMB ≥12 mut/Mb, 33.3% vs. 7.1%). Based on the results from the subgroup analysis in the KEYNOTE 061, the cutoff value of TMB-high and TMB-low was defined as 10 mut/Mb (191). Also, the TMB-high population had a better PFS and OS outcome (191). Similar to PD-L1, determining the threshold of TMB is crucial for its utilization as an alternative biomarker (192). In addition, the heterogeneity in tissue-based TMB and blood-based TMB also needs further larger-panel detection methods as well.

### 4.6 Circulating tumor cell and circulating tumor DNA

Minimal residual lesions can be detected through liquid biopsy, including circulating tumor cell (CTC) and circulating tumor DNA (ctDNA), which captured the recurrence clues earlier (193–197). Ying Jin et al. demonstrated that plasma ctDNA was correlated with ICI-induced resistance and corresponding AEs in GC (198). Also, several research studies emphasized the independent predicting value of CTC and ctDNA in GC immunotherapy, but low sensitivity and low accuracy, in part, restricted its promotion in clinical practice (198, 199). To solve this issue, novel PCR techniques [ddPCR; amplification refractory mutation system (ARMS) PCR; and breads, emulsification, amplification and magnetics (BEAMing)] and NGS-based methods have been exploited (194, 200–202).

### 4.7 Angiogenesis-related molecules (VEGF expression, angiogenic cytokines, and microvessel density)

Angiogenesis is one of the characteristics in cancer, and GC typically expresses high-level VEGF and secretes pro-angiogenic cytokines. It was reported that VEGF-D and VEGFR-3 could independently predict the poor prognosis after resection (203). Moreover, the researchers also found that patients with GC with lower microvessel density prolonged the survival (204). In a phase III and randomized trial (the AVAGAST), the baseline plasma VEGF-A expressions and lower-baseline neuropilin-1 levels both could be the potential biomarkers, which are correlated with the OS improvement with bevacizumab (205). At present, although anti-angiogenic drugs have shown potential and reliable safety in clinical trials, unique and appropriate biomarkers have not yet been established in GC.

### 4.8 Other novel molecules

The number and spatial distribution of effector T cells is another promising biomarker. Given the complexity of TME in GC, the scholars established one predictive model for immunotherapy response through multi-dimensional analysis. Taken together, signatures of infiltrating immune cells in tumor lesions favorably reflect as non-responder, low-responder, or high-responder. Intriguingly, CD8^+^ PD-1^+^ LAG-3^−^ cells and its spatial density contribute more among those studied immune cells (206). Furthermore, the investigators found that CLDN 18.2(+) tumors are rich in non-depleted CD8^+^ T cells (such as CD8^+^ PD-1^−^, CD8^+^ TIM3^−^, and CD8^+^ LAG3^−^ cell subtypes) (207). In addition, CD4^+^ FOXP3^−^ PD-L1^−^, and CD4^+^ FOXP3^−^ CTLA-4^−^ cells are also frequently detected. It may partly explain why CLDN 18.2–directed therapy and CLDN 18.2 CAR-T are highly effective.

## 5 Summary and future perspective

Even though there is a substantial breakthrough in advanced GC, more druggable targets and reliable biomarkers are still necessary (**Tables 3**, **4**). The combination of ICIs with chemotherapy has notably improved clinical outcomes in first-line treatments for GC/GEJC. However, the trials CheckMate 032 and CheckMate 649 showed that the combination of nivolumab and ipilimumab did not demonstrate improved efficacy in either first-line or second-line treatment settings (178, 209). Specifically, cohort 3 of the CheckMate 649 trial revealed that the combination of ipilimumab and nivolumab did not outperform chemotherapy; yet, a longer duration of response was noted in the dual-ICI arm, approximately double that of the chemotherapy arm. Hence, owing to its potential in reversing acquired chemotherapy resistance and the irreplaceable role of chemotherapy, the combined regimen of nivolumab, ipilimumab, and chemotherapy continues to be highly anticipated. Undoubtedly, adding ipilimumab may increase the risk of toxicities. Then, Cadonilimab (AK104), the only bispecific antibody targeting both PD-1 and CTLA-4, offers a unique mechanism by simultaneously blocking the interaction of PD-1 and CTLA-4 with their respective ligands, differing from the simple overlap of individual ICIs (210). The phase Ib/II trial, AK104–201, reported that AK104 in combination with XELOX (capecitabine + oxaliplatin) extended PFS and OS regardless of PD-L1 expression, with an acceptable safety profile (211). Among the 88 participants, 56 patients achieved partial response. Importantly, the proportion of patients exhibiting a PD-L1 CPS of ≥5 in this study was only 16.7%, markedly lower than that observed in the CheckMate 649 and ORIENT-16, where it was nearly 60%. Then, 2-year follow-up data updated that the ORR reached 68.2% after AK104 treatment; in detail, five patients reached complete response (5/88, 5.7%), and 55 patients reached partial response (55/88, 62.5%) (212).

**Table 3:** Current clinical trials about targeted agents in unresectable or metastatic gastric cancer.

Target	Clinical trial	Regimen	Agent	Line	Phase	Number	Endpoint	ORR (%)
HER-2	ToGA (60)	Trastuzumab + chemo vs. chemo	mAb	1L	III	594	OS, 13.8 vs. 11.1 months PFS, 6.7 vs. 5.5 months	47.3 vs. 34.5
	JACOB (62)	Chemo + trastuzumab + pertuzumab vs. Chemo + trastuzumab	mAb	1L	III	780	OS, 18.1 vs. 14.2 months PFS, 8.5 vs. 7.2 months	57.0 vs. 48.6
	LOGIC (61)	Lapatinib + chemo vs. chemo	mAb	1L	III	545	OS, 12.2 vs. 10.5 months PFS, 6.0 vs. 5.4 months	53.0 vs. 39.0
	TyTAN (63)	Lapatinib + paclitaxel vs. paclitaxel	mAb	2L	III	261	OS, 11.0 vs. 8.9 months PFS, 5.4 vs. 4.4 months	27.0 vs. 9.0
	GATSBY (66)	Trastuzumab-emtansine vs. docetaxel/paclitaxel	ADC	2L	II/III	345	OS, 7.9 vs. 8.6 months PFS, 2.7 vs. 2.9 months	20.6 vs. 19.6
	DESTINY-Gastric01 (67)	Trastuzumab-deruxtecan vs. irinotecan/paclitaxel	ADC	2L	II	188	OS, 12.5 vs. 8.4 months PFS, 5.6 vs. 3.5 months	51.0 vs. 14.0
VEGF/ VEGFR	RAINFALL (208)	Ramucirumab + chemo vs. chemo	mAb	1L	III	645	OS, 11.2 vs. 10.7 months PFS, 5.7 vs. 5.4 months	41.1 vs. 36.4
	REGARD (119)	Ramucirumab vs. placebo	mAb	2L	III	355	OS, 5.2 vs. 3.8 months PFS, 2.1 vs. 1.3 months	3.0 vs. 3.0
	RAINBOW (118)	Ramucirumab + paclitaxel vs. paclitaxel	mAb	2L	III	665	OS, 9.6 vs. 7.4 months PFS, 4.4 vs. 2.9 months	28.0 vs. 16.0
c-MET	RILOMET-1 (98)	Rilotumumab + chemo vs. chemo	mAb	1L	III	609	OS, 8.8 vs. 10.7 months PFS, 5.6 vs. 6.0 months	29.8 vs. 44.6
	METGastric (97)	Onartuzumab + FOLFOX vs. FOLFOX	mAb	1L	III	562	OS, 11.0 vs. 11.3 months PFS, 6.7 vs. 6.8 months	46.1 vs. 40.6
FGFR2	FIGHT (105)	Bemarituzumab + FOLFOX vs. FOLFOX	mAb	1L	II	155	OS, NR vs. 12.9 months PFS, 9.5 vs. 7.4 months	53.0 vs. 40.0
	SHINE (106)	AZD4547 vs. paclitaxel	TKI	2L	II	71	OS, 5.5 vs. 6.6 months PFS, 1.8 vs. 3.5 months	2.6 vs. 23.3
Claudin 18.2	FAST (131)	Zolbetuximab + chemo vs. chemo	mAb	1L	II	252	OS, 13 vs. 8.4 months PFS, 7.5 vs. 5.3 months	47.0 vs. 33.0

Chemo, chemotherapy; mAb, monoclonal antibody; ADC, antibody–drug conjugate; TKI, tyrosinase inhibitor; FOLFOX, 5-FU + leucovorin + oxaliplatin; PFS, progression-free survival; OS, overall survival; ORR, objective response rate; NA, not available; NR, not reached.

**Table 4:** Current clinical trials about ICI-based regimen in unresectable or metastatic gastric cancer.

Clinical trial	Regimen	Line	Phase	Number	OS (months)	PFS (months)
KEYNOTE 811 (155) (NCT03615326)	Pembrolizumab + trastuzumab + XELOX/PF vs. placebo + trastuzumab + XELOX/PF	1L	III	698	20.0 vs. 16.8 (HR = 0.84)	10.0 vs. 8.1 (HR = 0.73)
KEYNOTE 062 (67) (NCT02494583)	Pembrolizumab vs. pembrolizumab + CAPOX/FOLFOX vs. placebo + CAPOX/FOLFOX	1L	III	763	10.6 vs. 11.1 (HR = 0.91)	2.1 vs. 6.4 (HR = 1.66)
KEYNOTE 859 (152) (NCT03221426)	Pembrolizumab + CAPOX/PF vs. CAPOX/PF	1L	III	545	12.9 vs. 11.5 (HR = 0.78)	6.9 vs. 5.6 (HR = 0.76)
KEYNOTE 061 (163) (NCT02370498)	Pembrolizumab vs. paclitaxel	2L	III	592	9.1 vs. 8.3 (HR = 0.82)	1.5 vs. 4.1 (HR = 1.27)
CheckMate-649 (178) (NCT02872116)	Nivolumab + XELOX/FOLFOX vs. XELOX/FOLFOX	1L	III	955	14.4 vs. 11.1 (HR = 0.71)	7.7 vs. 6.05 (HR = 0.68)
ATTRACTION-04 (156) (NCT02746796)	Nivolumab + SOX/CAPOX vs. SOX/CAPOX	1L	II/III	724	17.45 vs. 17.15 (HR = 0.90)	10.45 vs. 8.34 (HR = 0.68)
ATTRACTION-2 (165) (NCT02267343)	Nivolumab vs. placebo	3L	III	493	5.32 vs. 4.14 (HR = 0.63)	NA
ORIENT-16 (158)	Sintilimab + CAPOX vs. CAPOX	1L	III	650	15.2 vs. 12.3 (HR = 0.76)	7.1 vs. 5.7 (HR = 0.636)
RATIONALE-305 (160)	Tislelizumab + XELOX/PF vs. XELOX/PF	1L	III	997	17.2 vs. 12.6 (HR = 0.74)	7.2 vs. 5.9 (HR = 0.67)
GEMSTONE-303 (161)	Sugemalimab + CAPOX vs. placebo + CAPOX	1L	III	479	15.64 vs. 12.45 (HR = 0.75)	7.62 vs. 6.08 (HR = 0.66)
JAVELIN Gastric 100 (162) (NCT02625610)	Avelumab maintenance vs. FOLFOX	–	III	805	10.4 vs. 10.9 (HR = 0.91)	NA
JAVELIN Gastric 300 (167) (NCT02625623)	Avelumab vs. paclitaxel/irinotecan	3L	III	371	4.6 vs. 5.0 (HR = 1.1)	1.4 vs. 2.7 (HR = 1.73)

FOLFOX, 5-FU + leucovorin + oxaliplatin; SOX, S-1 + oxaliplatin; CAPOX, cisplatin + capecitabine; 1L, first line; 2L, second line; 3L, third line; PFS, progression-free survival; OS, overall survival; HR, hazard ratio; NA, not available.

Meanwhile, data from real-world studies corroborate these above findings (213). Among the 22 patients with advanced GC/GEJC with PD-L1 CPS ≤5, 15 patients reached partial response, and 7 patients reached stable disease. Altogether, this suggests that cadonilimab may confer benefits even to those with low or negative PD-L1 expression levels. Motivated by the promising results of AK104, a phase III clinical trial (AK104–302) is currently underway. These endeavors provide fresh perspectives in the development of anti-tumor medicines through targeting distinct molecules. Similarly, ZW25 and KN026, which target bispecific, non-overlapping epitopes on HER-2, have shown superiority over conventional monoclonal antibodies. The efficacy of AMG 910 (a CD3/Claudin 18.2 bispecific antibody) and SPX-301 (a PD-L1/Claudin 18.2 bispecific antibody) in preclinical trials for GC has been validated.

ADCs are designed to deliver cytotoxic drugs directly to tumor cells, primarily leveraging the antibody-dependent cellular cytotoxicity effect. Instead, HER-2–directed ADCs alone have not yielded satisfactory outcomes. In a recent phase I trial, researchers investigated the efficacy and safety of combining Disitamab Vedotin (RC48) with toripalimab in GC/GEJC (214). This trial enrolled a total of 56 participants, with 30 cases of GC/GEJC. Among these, in the HER-2–positive cohort and the HER-2–low cohort, the ORR reached 56% and 46%, respectively. The potential of combining HER-2–targeted ADCs with ICIs in the second-line or even the first-line treatment deserves further explorations. Furthermore, targeting other immune checkpoints, such as LAG-3 and TIGIT, is also feasible and promising (145; 215, 216).

Apart from HER-2, other emerging targets (such as Claudin 18.2, FGFR, and c-MET) have shown their potential in advanced GC. Considering the heterogeneity among current trials, the future research studies require focusing on how to optimize the combination and sequence of immunotherapy and targeted treatments. Specifically, a reasonable combination could maximize the efficacy and, meanwhile, reduce adverse events. In addition, sequential therapies also contribute to overcome drug resistance. Moreover, novel therapeutic approaches such as CAR-T therapy (such as Claudin 18.2-specific CAR T cells) and tumor vaccine (such as neoantigen-loaded dendritic cell vaccine) are also promising owing to the efforts of translational studies in GC (115, 217). Taking the issues of HER-2–targeted agents that induced refractory as an example, small-molecule HER-2 inhibitor pyrotinib failed to achieve the expected efficacy in GC. To claim the cause, the scholars found that pyrotinib could upregulate the level of Cyclin D1 in HER-2–positive GC cells; furthermore, the addition of CDK4/6 inhibitors (SHR6390) could synergistically exert anti-tumor effect, which have been confirmed in preclinical AVATAR mice and a phase I clinical trial (NCT03480256) as well (218). In another preclinical study, the researchers reported the potential anti-angiogenesis role of Atractylenolide III (AT-III) via reducing microvessel density and HIF-1α in advanced GC (219, 220).

Merely focusing on anti-tumor drug development and updating is insufficient for advanced GC management. Greater emphasis should be placed on selecting appropriate candidates for treatment. Hence, identifying reliable biomarkers is crucial for precision medicine (221, 222). Current markers such as PD-L1 positivity, MSI-high status, high tumor mutational burden, and EBV infection do not fully meet the requirements. Establishing a multi-biomarker network and framework that incorporate these molecules and consider the heterogeneity of gastric and gastroesophageal tumors, along with HER-2 status, c-MET, or FGFR, would be instrumental in differentiating between non-responders, low-responders, and high-responders to chemotherapy, immunotherapy, or molecular targeted therapy. Regarding liquid biopsies (whether NGS-based or PCR-based techniques), standardization and optimization detection procedures via leveraging the emerging omics methods is another critical aspect.

Ultimately, precision and personalization have become paramount in the treatment of unresectable or metastatic GC. The exploration and integration of existing therapeutic targets, along with in-depth research into novel molecules, warrant further investigation in the future.

## Author contributions

ZZ: Writing – review & editing, Writing – original draft, Investigation. QZ: Writing – review & editing, Validation, Funding acquisition, Conceptualization.
